# Retrospective identification of medication related adverse events in the emergency medical services through the analysis of a patient safety register

**DOI:** 10.1038/s41598-022-06290-9

**Published:** 2022-02-16

**Authors:** Ian Howard, Ian Howland, Nicholas Castle, Loua Al Shaikh, Robert Owen

**Affiliations:** Hamad Medical Corporation Ambulance Service, Hamad Medical Corporation, Doha, Qatar

**Keywords:** Outcomes research, Epidemiology

## Abstract

Adverse drug events encompass a wide range of potential unintended and harmful events, from adverse drug reactions to medication errors, many of which in retrospect, are considered preventable. However, the primary challenge towards reducing their burden lies in consistently identifying and monitoring these occurrences, a challenge faced across the spectrum of healthcare, including the emergency medical services. The aim of this study was to identify and describe medication related adverse events (AEs) in the out-of-hospital setting. The medication components of a dedicated patient safety register were analysed and described for the period Jan 2017–Sept 2020. Univariate descriptive analysis was used to summarize and report on basic case and patient demographics, intervention related AEs, medication related AEs, and AE severity. Multivariable logistic regression was used to assess the odds of AE severity, by AE type. A total of 3475 patient records were assessed where 161 individual medication AEs were found in 150 (4.32%), 12 of which were categorised as harmful. Failure to provide a required medication was found to be the most common error (1.67%), followed by the administration of medications outside of prescribed practice guidelines (1.18%). There was evidence to suggest a 63% increase in crude odds of any AE severity [OR 1.63 (95% CI 1.03–2.6), p = 0.035] with the medication only AEs when compared to the intervention only AEs. Prehospital medication related adverse events remain a significant threat to patient safety in this setting and warrant greater widespread attention and future identification of strategies aimed at their reduction.

## Introduction

An adverse drug event (ADE) is defined as "*an injury resulting from medical intervention related to a drug"* and encompasses a wide range of potential unintended and harmful events from adverse drug reactions to medication errors^1^. ADEs can occur in any location or setting in the continuum of care of a patient and are estimated to account for as many as 1 in 3 of all in-hospital adverse events, affecting approximately 2 million hospital stays each year, where they prolong hospital stays by between 1.7 and 4.6 days^2^. Each year, ADEs in the outpatient setting account for over 3.5 million physician office visits, approximately 1 million emergency department (ED) visits, and approximately 125,000 hospital admissions^2^. These outpatient presentations will not only spend more days in hospital but incur nearly double the healthcare costs compared with patients presenting without medication related morbidity^3^. Despite this significant burden, many of these events are, in retrospect, considered preventable^4,5^. However, the primary challenge towards reducing this burden lies in consistently identifying and monitoring these occurrences, a challenge faced across the spectrum of healthcare, including the emergency medical services.

The Emergency Medical Services (EMS) represent a considerable and increasingly essential entry point into the healthcare system across the world. Patients utilising these services are often amongst the most critical, with many requiring initial intervention or resuscitation prior to reaching hospital^6–12^. Given the emergent nature in which these patients present to EMS, coupled with the fact that these services are often provided in the backdrop of challenging environments, with few diagnostic resources available, and for patients of varying acuity, the potential for and adverse drug events (ADEs) to occur is significant. Despite this potential, the scientific literature regarding the occurrence or reporting of ADEs in the EMS setting is severely lacking.

There are likely several factors that have affected this, starting with the traditional focus of EMS clinical governance activities on operational and/or intervention related activities such as cardiopulmonary resuscitation (CPR), defibrillation or endotracheal intubation^6^. This has been further confounded by the significant international variation in EMS service provision, staff training and experience, and scope of practice^7–12^. Lastly, much of the literature that has been historically reported was limited to the identification of rates of self-reported ADEs or small sample descriptive analyses^13–17^. Consequently, reported rates of medication related AEs within the EMS setting have varied considerably and understanding their true remains largely unknown. As a result, the aim of this study was to comprehensively identify and describe medication related AEs in the EMS setting, and to compare these event rates with intervention related AEs, the traditional focus for AE detection in EMS clinical governance.

## Methods

A descriptive observational analysis of a patient safety registry was conducted in order to achieve the study aims and objectives.

### Setting

The study was conducted within the Hamad Medical Corporation Ambulance Service (HMCAS), the national ambulance service of the State of Qatar. Qatar is a country located in West Asia, situated on the Qatar peninsular in the Persian Gulf, with a population of approximately 3 million people. As is common in the region, the majority of the population of Qatar is composed of expatriate residents, with Qatari citizens making up approximately 15%–20% of the population. HMCAS is a two-tiered emergency medical service provider with Ambulance Paramedic (AP) staffed ambulances and advanced Critical Care Paramedic (CCP) staffed fast-response vehicles. On average, approximately 75 ambulances, seven CCP fast-response vehicles and two helicopters are in operation per shift, and service an average daily call rate of approximately 1000–1200 community cases (70%) and inter-facility transfers (30%).

### Method

The participating service maintains a patient safety registry, where a sample of patients treated and transported by the critical care division and helicopter emergency medical service (HEMS) division are randomly extracted on a monthly basis and reviewed using an established methodology for detecting AEs in the EMS setting. The registry employs the use of a novel approach, known as the "trigger tool" (TT) methodology, which has seen some success towards the investigation of both general and drug related adverse events (AEs) in the inhospital and prehospital emergency setting^18–30^. The TT methodology is the application of a retrospective sampling framework that allows for the detection and targeted identification of specific cases at greatest risk for a potential AE (unintended consequence associated with medical care) and harm (injury or illness resulting from or contributed to by such occurrences). This is accomplished through the recognition of abnormal or unexpected values, measurements, notes or 'rules' for any given medical record. The aim of the TT methodology is to evaluate a defined sample of patients to determine whether or not an AE and patient harm are present, and to measure the rates over time as improvement work focuses on the reduction of such events^29^. Use of the TT methodology is reported to provide a more time-effective, cost-effective, and sensitive means of identifying AEs and harm when compared with traditional methods, such as conventional chart review or voluntary reporting^25–32^. The specific trigger tool used by the registry is a modified version of the Pitt AE trigger tool, a previously validated prehospital specific trigger tool with a focus on high-risk case types^22^. There are 11 sections in the tool and registry, three of which focus on medication related AEs (Table 1). Following an internal validation process, additional high-risk medications, and interventions specific to the local setting were introduced to the tool and tested over a 6-month period prior to full implementation. For the purposes of this study, the intervention and medication related sections of the registry were described.

**Table 1:** TT methodology audit items.

Code	Description
**T8 descriptors: failure of any intervention/procedure during patient care**	
8.1	Multiple IV attempts
8.2	Failed IV
8.3	Failed IO
8.4	Failed external jugular cannulation
8.5	Failed NGT
8.6	Failed electrical cardioversion
8.7	Failed defibrillation
8.8	Failed transcutaneous pacing
8.9	Failed ETI
8.10	Failed LTA
8.11	Failed surgical airway
8.12	Failed needle decompression
8.13	Failed finger thoracostomy
8.14	Failed mechanical ventilator
8.15	Other
**T9 descriptors: use of the following medications**	
9.1	Adrenaline
9.2	Phenylephrine
9.3	Noradrenaline
9.4	Naloxone
9.5	Rocuronium
9.6	Fentanyl
9.7	Ketamine
9.8	Midazolam
9.9	TXA
9.10	Amiodarone
9.11	Adenosine
**T10 descriptors: any deviation from CPGs**	
10.1	Intervention outside CPG
10.2	Medication outside CPG
10.3	Failure to provide required medication
10.4	Failure to provide required intervention
10.5	Other
**T11 descriptors: medication error**	
11.1	Wrong medication administered
11.2	Wrong dose administered
11.3	Administered via wrong route
11.4	Other

*AE* adverse event; *IV* intravenous; *IO* intraosseous; *NGT* nasogastric tube; *ETI* endotracheal intubation; *LTA* laryngeal tube airway; *CPG* clinical practice guidelines.

**Figure 1:**
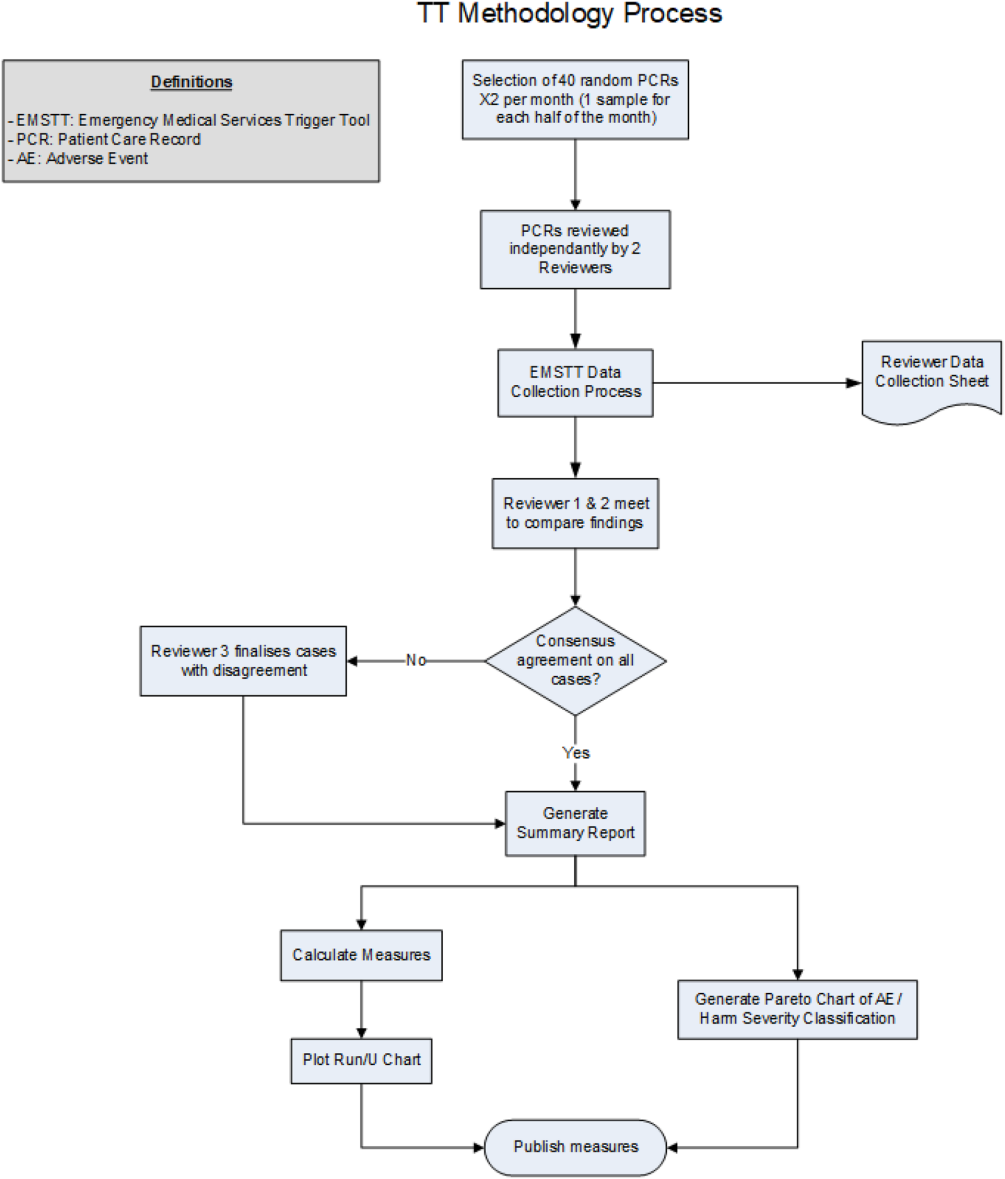
Trigger tool methodology process (AE: Adverse event; EMSTT: Emergency Medical Services Trigger Tool; PCR: Patient Care Report).

For the register, a random sample of patient care records from each participating service division are selected each month and independently reviewed by two primary reviewers. The primary reviewers are operational CCPs within each of the divisions that the patient care records (PCRs) are extracted from. Prior to utililsation, reviewers are given a brief didactic lecture on the concept of trigger tools as well as the applied trigger tool, and its application to 10 test cases. Following this, the reviewers are tested on a live monthly sample and compared with the output from current reviewers. Following each review round, the two primary reviewers meet to compare findings, reach consensus, and summarize the results. In cases where consensus could not be reached, a third reviewer, was consulted to determine an outcome (Fig. 1). Each record was manually reviewed for the presence of an AE trigger only. If a trigger was found, the record was further reviewed for the occurrence of harm. Records that did not contain a trigger were not reviewed further. For cases where an AE was found, an AE severity was assigned by the reviewers (Table 2). Given the limited amount of time with which patients are exposed to EMS, assigning a severity was initially limited to three categories: AE with evidence of harm, AE with the potential to cause harm, and no AE. In addition, due to these time-based constraints, and given limitations in prehospital EMS documentation, a formal causality assessment such as the WHO-UMC system could not be conducted and consequently was not part of the analysis. Beginning in 2018 an additional, EMS specific severity classification system developed by Patterson et al.^23^, the Adverse Event Severity Rating Index, was introduced to run concurrently with the three-category system (Table 2). This allowed a fit for purpose EMS-specific system to be used for AE severity classification, and to allow for greater descriptive detail.

**Table 2:** Proximal cause and severity definitions.

Proximal cause		
Category	Description	Definition
1	Actions by patient	The AE was the result of action(s) by the patient
2.1	Actions by provider—CCP crew	The AE was the result of action(s) or inaction(s) by the crew
2.2	Actions by provider—Non CCP crew	
3.1	Medical or vehicle equipment—CCP crew	Failure of the equipment, failure to troubleshoot and correct common problems with the equipment, or failure to remove defective equipment from service
3.2	Medical or vehicle equipment—non CCP crew	
4	Environmental/scene factors	Factors that may result from weather conditions or factors on the ground/scene (or other). This includes temperature, light, and scene safety
5	Undetermined by chart review	The proximal cause of the AE (regardless of severity) cannot be determined by the information available in the chart

Category	Description	
**Severity classification system 1**		
AE1	AE with evidence of patient harm	
AE2	AE with potential to cause harm	
AE3	No evidence of harm	
**Severity classification system 2**		
S1	AE with harm as a result of commission	
S2	AE with harm as a result of omission	
S3	AE with harm, but no fault	
S4	AE with potential to cause harm as a result of commission	
S5	AE with potential to cause harm as a result of omission	
S6	AE with potential to cause harm with no fault	
S7	No harm identified	

*AE* adverse event; *CCP* critical care paramedic.

### Sample size

For the purpose of this study, all registry data collected from Jan 2017 (the initiation of the registry) to Sept 2020 was included in the analysis.

### Variables

The primary exposure under assessment was the occurrence of medication related AEs in the patient safety register and was ascertained for each case by the record reviewers based on the details documented in the patient care record. The primary outcome under assessment was the occurrence of assigned AE severity types and was too ascertained for each case by the record reviewers. Secondary exposures and outcomes under assessment included the occurrence of intervention related AEs, and their assigned AE severity category. As with the medication AEs, intervention related exposures and outcomes were ascertained by the record reviewers. Given that a single patient could experience both an intervention AE and a medication AE simultaneously, for the purposes of the multivariable analysis, records where an AE occurred was extracted and categorised into medication AE only; intervention AE only; and combined medication AE + intervention AE. For this analysis, the intervention AE group was used as the reference group.

Due to the operational focus of the patient safety registry, in-depth data regarding confounding variables were not routinely captured. However, for the purposes of this study, gender, age, and broad case type were considered a priori as potential confounders in the relationship between AE occurrence and AE severity and were included in the analysis as and where necessary.

### Statistical analysis

Univariate descriptive analysis was be used to summarize and report on basic case and patient demographics, intervention related AEs, medication related AEs, and AE severity. Counts and proportions were used to describe all summarized univariate analysis. Chi-square analysis was used as the primary measure of significance for categorical data, the unpaired two-sample t-test for comparisons of means of continuous data, and the two-sample z-test of proportions for categorical data as required. 95% confidence intervals were calculated where necessary and a p-value of 0.05 used as a cut-off for statistical significance. Multivariable logistic regression was used to assess the odds of the primary outcomes (AE severity category), by exposure type (AE type), adjusting for multiple variables of interest. Crude and adjusted odds and odds ratios (OR) were used as the primary measure of effect for the multivariable analysis and reported with 95% C.I.s. For the multivariable analysis, two models were constructed for each outcome of interest: one for the crude association with exposure, and a second model adjusted to include the a priori confounders, to identify their influence, and to assess the true underlying effect of the intervention. All statistical analyses were performed using Stata version 15.1 (Stata Corporation, College Station, TX, USA).

## Results

A total of 3475 patient records were available in the registry and included in the study. The majority of patients were male (79.17%), aged between 45 and 54 (19.24%) and had an underlying medical reason for utilising EMS (65.84%) (Table 3). A total of 161 individual medication related errors were recorded, amongst 150 patients in the registry (4.32%). Failure to provide a required medication was the most common (1.67%), followed by the administration of medications outside of those prescribed by clinical practice guidelines (CPGs) (1.18%), and medications in which the wrong dose was administered (1.12%). A total of 139 patients experienced a single medication AE, with a further 11 patients experiencing multiple medication AEs. Amongst the medications of interest that were monitored, Fentanyl was the most frequently administered (27.31%), followed by Amiodarone (22.83) and Adrenaline (21.27%).

**Table 3:** Demographic and descriptive data.

Characteristics	Total patients		P value*
	N	%	
Total patients	3475		
**Gender**			
Male	2751	79.17	0.057
Female	724	20.83	
**Age category**			
< = 14	72	2.14	0.199
15–24	366	10.87	
25–34	651	19.33	
35–44	591	17.55	
45–54	648	19.24	
55–64	431	12.80	
65–74	300	8.91	
> = 75	309	9.17	
Missing	107	3.08	
**Case type**			
Medical	2288	65.84	0.508
Trauma	1187	34.16	
**Intervention related AEs**			
Any intervention AE	296	8.52	
Multiple IV attempts	93	2.68	
Failed IV	77	2.22	
Failed IO	5	0.14	
Failed external jugular cannulation	2	0.06	
Failed NGT	1	0.03	
Failed electrical cardioversion	0		
Failed defibrillation	0		
Failed transcutaneous pacing	4	0.12	
Failed ETI	41	1.18	
Failed LTA	13	0.37	
Failed surgical airway	0		
Failed needle decompression	4	0.12	
Failed finger thoracostomy	0		
Failed mechanical ventilator	13	0.37	
Other significant intervention failure	16	0.46	
Failure to provide required intervention	46	1.32	
Intervention outside CPG	30	0.86	
Failure of other intervention-based process	19	0.55	
**Multiple intervention related AEs**			
0 AEs	3179	91.48	
1 AE	237	6.82	
2 AEs	51	1.47	
3 AEs	7	0.20	
4 AEs	1	0.03	
**Medication related AEs**			
Any medication related AE	150	4.32	
Medication outside CPG	41	1.18	
Failure to provide required medication	58	1.67	
Wrong medication administered	9	0.26	
Wrong dose administered	39	1.12	
Administered via wrong route	2	0.06	
Other medication error	8	0.23	
Failure of other medication-based process	4	0.12	
**Multiple medication related AEs**			
0 AEs	3325	95.68	
1 AE	139	4.00	
2 AEs	11	0.32	
**Administration of the following medication of interest**			
Adrenaline	739	21.27	
Phenylephrine	142	4.09	
Noradrenaline	3	0.09	
Naloxone	13	0.37	
Rocuronium	380	10.94	
Fentanyl	949	27.31	
Ketamine	657	19.91	
Midazolam	173	4.98	
Tranexamic acid	182	5.68	
Amiodarone	732	22.83	
Adenosine	64	2.00	

*AE* adverse event; *IV* intravenous; *IO* intraosseous; *NGT* nasogastric tube; *ETI* endotracheal intubation; *LTA* laryngeal tube airway; *CPG* clinical practice guidelines.

*Pearson's chi-squared test for association with medication related AEs.

As the only potential confounding variables captured on the registry and included in the study, we evaluated the relationship between medication related AEs and age, gender, and case type (Table 3). There was little evidence to suggest a relationship between either age (p = 0.199) or case type (p = 0.508) with the occurrence of any medication related AE, yet some evidence to suggest a relationship with gender (p = 0.057). Despite these results, prior to the analysis, each of these variables were considered as a priori confounding variables and nonetheless still included in the multivariable analysis as such.

From an AE severity rating perspective, of key interest were the categories in which there was demonstrable evidence of patient harm. Consequently, the proportion of patients with an AE, and the proportion of these AEs classified as resulting in patient harm were compared between intervention and medication related AEs (Table 4). As mentioned above, there was a higher proportion of patients who experienced an intervention related AE (0.0852), compared with a medication AE (0.0432), with evidence to suggest a difference between the two (< 0.0001). Despite the difference, this is likely as a result of the higher number of intervention "triggers" in the review tool, compared to medication related "triggers". In terms of AE severity rating, it is of interest to note that there was a marginally higher nominal proportion of intervention related AEs with the broader severity classification of AE1—*AE with evidence of patient harm*, (0.0961) compared with medication AEs (0.0745). However, when compared with the newer more "in-depth" severity classification system, a higher proportion of medication AEs were experienced across these (S1–S3) compared with intervention AEs.

**Table 4:** Comparison of proportion of AEs and AE severity classification.

Characteristic	Numerator	Denominator	Proportion	P value*
**Patients with an AE**				
Any intervention AE	296	3475	0.0852	< 0.0001
Any medication AE	150	3475	0.0432	
**Severity classification AE1**				
Any intervention AE	35	364	0.0961	0.424
Any medication AE	12	161	0.0745	
**Severity classification S1**				
Any intervention AE	2	313	0.0064	0.398
Any medication AE	2	138	0.0145	
**Severity classification S2**				
Any intervention AE	3	313	0.0036	0.199
Any medication AE	2	138	0.0145	
**Severity classification S3**				
Any intervention AE	8	313	0.0256	0.058
Any medication AE	0	138	0	

*Z test for comparison of proportions where diff ! = 0.

Despite the marginal nominal difference in proportions between the groups, there was no evidence to suggest a statistical difference in proportions of medication AEs compared with intervention AEs for those classified as AE1—*AE with evidence of patient harm* (p = 0.424), S1—*AE with harm as a result of commission* (p = 0.398), or S2—*AE with harm as a result of omission* (p = 0.199). In terms S3—*AE with harm, but no fault*, there was some evidence to suggest a statistical difference in proportions (p = 0.058).

The different subtypes of AEs and their resultant severity classification were explored in greater detail to further understand these individual occurrences (Table 5). Amongst the 161 individual medication related AEs, 12 (7.45%) were rated as AE1—*AE with evidence of patient harm*, with wrong medication dose the most common (n = 11, 28.21%). As with the intervention AEs, for the newer severity classification, AEs were rated more conservatively, with only 2 AEs classified as S1—*AE with harm as a result of commission* (1.45%), 4 cases classified as S2—*AE with harm as a result of omission* (2.90%), and 0 cases classified as S3—*AE with harm, but no fault*. Amongst the medications of interest documented in the registry, Tables 6 and 7 describes the occurrence of medication related AE type and severity by medication type administered. In terms of severity classification, Rocuronium was the medication found to be most commonly reported with a severity classification of AE1—*AE with evidence of patient harm*, (n = 22, 5.79%), followed by Adrenaline (n = 12, 1.62). In contrast, with the new severity classification system, both Adrenaline and Amiodarone were more commonly reported for severity classification S1—*AE with harm as a result of commission* (n = 2, 0.27%), S2—*AE with harm as a result of omission* (n = 2, 0.27%), and S3—*AE with harm, but no fault* (n = 6, 0.82%).

**Table 5:** AE severity categories by AE type.

Characteristic	Total AEs N (%)	AE1 N (%)	AE2 N (%)	Total N (%)	S1 N (%)	S2 N (%)	S3 N (%)	S4 N (%)	S5 N (%)	S6 N (%)
	Severity classification 1			Severity classification 2						
Medication related AEs										
Any medication related AE	161	12 (7.45)	31 (19.25)	138	2 (1.45)	4 (2.90)	0	46 (33.33)	37 (26.81)	12 (8.70)
Medication outside CPG	41	0	1 (2.44)	36	0	0	0	26 (72.22)	1 (2.78)	1 (2.78)
Failure to provide required medication	58	1 (1.72)	0	53	0	3 (5.66)	0	2 (3.77)	26 (49.06)	10 (18.87)
Wrong medication administered	9	0	5 (55.56)	8	0	0	0	3 (37.50)	1 (12.50)	1 (12.50)
Wrong dose administered	39	11 (28.21)	19 (48.72)	27	1 (3.70)	0	0	12 (44.44)	6 (22.22)	0
Administered via wrong route	2	0	1 (50.00)	2	0	0	0	1 (50.00)	0	0
Other medication error	8	1 (12.50)	4 (50.00)	8	1 (12.50)	0	0	1 (12.50)	3 (37.50)	0
Failure of other medication-based process	4	0	1 (25.00)	4	0	1 (25.00)	0	1 (25.00)	0	0

AE—adverse event; CPG—clinical practice guideline.

AE1—AE with evidence of patient harm.

AE2—AE with potential to cause harm.

S1—AE with harm as a result of commission.

S2—AE with harm as a result of omission.

S3—AE with harm, but no fault.

S4—AE with potential to cause harm as a result of commission.

S5—AE with potential to cause harm as a result of omission.

S6—AE with potential to cause harm with no fault.

**Table 6:** Medication of interest by medication AE.

Characteristic	Total patients receiving	Any medication related trigger	Medication outside CPG	Failure to provide required medication	Wrong medication administered	Wrong dose administered	Administered via wrong route	Other medication error	Failure of other medication-based process
	N	N (%)	N (%)	N (%)	N (%)	N (%)	N (%)	N (%)	N (%)
Adrenaline	739	32 (4.33)	5 (0.68)	12 (1.62)	2 (0.27)	7 (0.95)	0	4 (0.54)	2 (0.27)
Phenylephrine	142	12 (8.45)	1 (0.70)	5 (3.52)	0	4 (2.82)	0	2 (1.41)	0
Noradrenaline	3	1 (33.33)	0	1 (33.33)	0	1 (33.33)	0	0	0
Naloxone	13		0	0	0	0	0	0	0
Rocuronium	380	26 (6.84)	3 (0.79)	8 (2.11)	2 (0.53)	11 (2.89)	0	1 (0.26)	1 (0.26)
Fentanyl	949	42 (4.43)	12 (1.26)	14 (1.48)	2 (0.21)	9 (0.95)	0	4 (0.42)	1 (0.11)
Ketamine	657	29 (4.41)	4 (0.61)	13 (1.98)	1 (0.15)	8 (1.22)	0	2 (0.30)	1 (0.15)
Midazolam	173	3 (1.73)	0	2 (1.16)	1 (0.58)	0	0	0	0
Tranexamic acid	182	7 (3.85)	6 (3.3)	0	0	1 (0.55)	0	0	0
Amiodarone	732	31 (4.23)	5 (0.68)	12 (1.64)	2 (0.27)	6 (0.82)	0	4 (0.55)	2 (0.27)
Adenosine	64	1 (1.56)	0	0	0	0	1 (1.56)	0	0

**Table 7:** AE severity categories by medication of interest.

Characteristic	Total patients N (%)	AE1 N (%)	AE2 N (%)	Total N (%)	S1 N (%)	S2 N (%)	S3 N (%)	S4 N (%)	S5 N (%)	S6 N (%)
	Severity classification 1			Severity classification 2						
Adrenaline	739	12 (1.62)	85 (11.50)	732	2 (0.27)	2 (0.27)	6 (0.82)	9 (1.23)	11 (1.50)	67 (9.15)
Phenylephrine	142	5 (3.52)	15 (10.56)	137	1 (0.73)	1 (0.73)	0	3 (2.19)	2 (1.46)	10 (7.30)
Noradrenaline	3	1 (33.33)	0	3	0	1 (3.33)	0	0	0	0
Naloxone	13	0	0	3	0	0	0	0	0	0
Rocuronium	380	22 (5.79)	43 (11.32)	285	1 (0.35)	0	0	6 (2.11)	6 (2.11)	32 (11.23)
Fentanyl	949	3 (0.32)	64 (6.74)	942	1 (0.11)	1 (0.11)	0	16 (1.70)	13 (1.38)	37 (3.93)
Ketamine	657	3 (0.46)	65 (9.89)	653	1 (0.15)	1 (0.15)	1 (0.15)	11 (1.68)	9 (1.38)	46 (7.04)
Midazolam	173	1 (0.58)	2 (1.16)	159	0	0	0	1 (0.63)	0	1 (0.63)
Tranexamic acid	182	0	11 (6.04)	182	0	0	0	5 (2.75)	0	6 (3.30)
Amiodarone	732	10 (1.37)	85 (11.61)	732	2 (0.27)	2 (0.27)	6 (0.82)	9 (1.23)	5 (1.50)	67 (9.15)
Adenosine	64	0	1 (1.56)	64	0	0	0	1 (1.56)	0	0

Lastly, a multivariable analysis was conducted, assessing the odds of AEs demonstrating patient harm, by broad AE type (Table 8). Model 1 analysed the odds of the AE types resulting in the broad severity classification AE1—*AE with evidence of patient harm*. There was however no evidence to support a crude [OR 1.14 (95% CI: 0.60–2.16), p = 0.700] or adjusted [OR 0.95 (95% CI: 0.47–1.93), p = 0.896] difference in OR between intervention only AEs and medication only AEs. Model 2 analysed the odds of any AE severity category by combining the severity categories AE1—*AE with evidence of patient harm* with AE2—AE *with potential to cause harm*. From this perspective, there was evidence to suggest a 63% increase in crude odds of any AE severity [OR 1.63 (95% CI: 1.03–2.6), p = 0.035] with the medication only AEs compared to the intervention only AEs. This increased remained after adjusting for confounders, albeit with limited statistical evidence supporting the increased association [OR 1.57 (95% CI: 0.97–2.54), p = 0.066].

**Table 8:** Multivariable analysis of AE severity category and AE type.

Characteristic	Total N (%)	Events N (%)	Crude OR (95% CI)	P value	Adjusted OR (95% CI)*	P value
**Model 1**						
Intervention AE only	279	30 (10.75)	1		1	
Medication AE only	133	16 (12.03)	1.14 (0.60–2.16)	0.700	0.95 (0.47–1.93)	0.896
Medication AE + intervention AE	17	5 (29.41)	**3.46 (1.13–10.49)**	**0.028**	2.82 (0.80–10.00)	0.107
**Model 2**						
Intervention AE only	279	174 (62.37)	1		1	
Medication AE only	133	97 (72.93)	**1.63 (1.03–2.56)**	**0.035**	**1.57 (0.97–2.54)**	**0.066**
Medication AE + intervention AE	17	14 (82.35)	2.82 (0.79–10.03)	0.110	2.83 (0.77–10.48)	0.118
**Model 3**						
Intervention AE only	234	11 (4.70)	1		1	
Medication AE only	116	5 (4.31)	0.91 (0.31–2.69)	0.869	0.99 (0.29–3.39)	0.987
Medication AE + intervention AE	13	1 (7.69)	1.69 (0.20–14.19)	0.629	1	
**Model 4**						
Intervention AE only	234	155 (66.24)	1		1	
Medication AE only	116	86 (74.14)	1.46 (0.89–2.40)	0.134	1.40 (0.82–2.39)	0.224
Medication AE + intervention AE	13	10 (76.92)	1.70 (045–6.35)	0.431	1.33 (0.33–5.26)	0.688

Model 1: AE 1 only.

Model 2: AE 1 + AE2.

Model 3: S1 + S2 + S3.

Model 4: S1 + S2 + S3 + S4 + S5 + S6.

*Adjusted by age category, gender, case type.

AE1—AE with evidence of patient harm.

AE2—AE with potential to cause harm.

S1—AE with harm as a result of commission.

S2—AE with harm as a result of omission.

S3—AE with harm, but no fault.

S4—AE with potential to cause harm as a result of commission.

S5—AE with potential to cause harm as a result of omission.

S6—AE with potential to cause harm with no fault.

Models 3 and 4 repeated the above analysis, utilising the newer more "in-depth" severity classification system. Model 3 combined the severity categories which demonstrated patient harm only yet found no difference in either crude [OR 0.91 (95% CI: 0.31–2.69), p = 0.869] or adjusted odds [OR 0.99 (95% CI: 0.29–3.39), p = 0.987] between intervention only AEs and medication only AEs. Model 4 combined all categories of severity categories including those with evidence of patient harm, and those with the potential for patient harm. However, as with Model 3, there was no evidence to suggest a difference in crude or adjusted odds between intervention only AEs and medication only AEs.

Of interest to note, across all models, patients with a combination of intervention and medication related AEs showed a general increase in odds for severity categories demonstrating patient harm and/or the potential for patient harm. However, the estimate is likely unreliable and hampered by poor precision given the small number of occurrences and events in this category.

## Limitations

Despite the strength of the methodology highlighted above, there were arguably several limitations that could equally be attributed to it. Firstly, and from a more generic perspective the data utilised for the study was not collected for the purpose of this study, and consequently there were likely data fields missing that would have been included had the data been prospectively collected. This is of particular importance regarding confounding variables, which was evident in this study where just three data fields were available to be used as potential confounders.

In addition, the data collected for the registry was collected retrospectively from case records where data quality was not measured or assessed. Consequently, the potential exists that poor documentation quality could have impacted the results given that AE occurrence was primarily determined by the case reviewers directly from the case records.

Similarly in terms of AE severity classification, these outcomes were primarily ascertained by the case reviewers, and arguably with significant subjective influence, given the loose severity classification criteria. This was evident in the difference in the results between the two severity classification systems applied, which the reviewers have commented on was somewhat improved with the introduction of the second classification system and its criteria that were more explicit in their definitions.

## Discussion

Given the short duration of time patients are exposed to EMS, and the limitations in the availability of diagnostic resources, much of the focus for the delivery of prehospital emergency care is concentrated on symptomatic management or processes of care, as opposed to outcomes. Consequently, much of the historical focus for improvement has concentrated on interventions such as cardiopulmonary resuscitation (CPR), defibrillation or endotracheal intubation. Furthermore, the scope of practice for the use of medications by EMS varies significantly across the globe. As a result, little is known regarding the occurrence of ADEs in the prehospital EMS setting. Of the literature that is available, much is limited to the self-reporting and/or adhoc analysis of a single patient sample.

In attempt to further understand the burden of ADEs in the EMS setting, we applied a novel methodology to a large sample dataset in order to better understand their occurrence. A total of 3475 patients were included in the analysis making this one of the largest studies dedicated to understanding ADEs in the prehospital environment, to date. We observed an absolute occurrence of medication related ADEs in 4.32% of patient records assessed, and 7.45% of all medication administrations, with failure to provide a required medication found to be the most common, followed by the administration of medications outside of those prescribed by clinical practice guidelines, and medications in which the wrong dose was administered. Comparison of the results of this study, with the literature is difficult given the paucity of literature available and variations in methodologies adopted.

Nonetheless, of the limited quantitative observational available the rate observed in our study was lower than that reported by Lifshitz et al.^15^ who described an ADE incidence of 12.76% in 188 patients, and 7.12% of the 435 drug administrations. Similarly, the types of ADEs observed in our study were too lower than related types observed by Lishitz, which included errors involved ordering a drug that was not indicated for the patient's condition in 32.6% of administrations or ordering an incorrect dose of the drug in 38.7% of administrations. Similarly, the rates of ADEs observed by Hoyle et al.^14^ in their retrospective patient record review were too higher than those observed in our study, with ADEs occurring in 125 of 360 doses administered (34.7%). Furthermore, Hoyle found Epinephrine to be amongst the drugs associated with the highest proportion of ADEs (65.1%), an observation not too dissimilar to our study where Adrenaline was a commonly associated with several ADEs. Of the self-reported ADE literature, Vilke observed ADE occurrences in type of medications not too dissimilar to that observed in this study, with Atropine, Epinephrine-1000 and Morphine found to be the most frequently reported drugs involving ADEs, compared to our study in which Adrenaline and Fentanyl were amongst the most frequent involving ADEs^13^. Similarly, there is some overlap regarding the underlying factors cited in contributing towards ADEs in the Vilke study compared with our study, with dosage calculation error, and incorrect dosage given being approximately shared between the two studies as common factors.

The significant differences observed between the results across the literature, and additionally compared with the results of this study are likely down to differences in methodology applied. From a broader perspective, there is a general lack of consistency in and guidelines towards the ideal method for identifying ADEs in the prehospital setting. Despite this, the TT methodology remains a promising potential given its focus on iterative sampling, rapid record review, and consensus exposure and outcome identification and classification. It therefore remains a potential for future research in the subject area. Similarly, the AE types, and severity classification are key to the successful application of the TT methodology and identification of the most appropriate and relevant categories too remains an area for future research.

This study did not attempt to specifically investigate the occurrence of ADRs in the prehospital setting. However, given the limited amount of time in which patients are exposed to these services, combined with the often-limited scope of medication used in the prehospital setting, and limitation in diagnostic resources available, this remains a difficult and unlikely avenue for future research. As a subset however, there remains potential to further understand the utilisation of EMS in the treatment and transportations of patients who have already experienced an ADR as a result of their ongoing and/or chronic medication, such as that conducted by Delhours et al.^16^. Such research further aides to comprehensively identify the burden exerted by this poorly understood issue, and as such remains a credible avenue for future research.

## Conclusion

Detecting and monitoring ADEs, in any setting, is a key component towards the development of strategies aimed at their reduction. The occurrence of ADEs in the prehospital setting is poorly understand, largely owing to the lack of scientific research, and lack of consistency in which this topic has been previously investigated and reported. The trigger tool remains a promising method towards achieving this. When applied to a large sample patient safety registry with a focus on ADEs, considerable insight can be gained towards understanding this burden. The occurrence of ADEs in the prehospital setting resulted in a higher proportion of cases with demonstrated patient harm compared to prehospital interventions, the historical focus for adverse event detection in the prehospital setting. Furthermore, the occurrence is higher than that reported in the ED using similar methods. Prehospital ADEs therefore remain a significant threat to patient safety in this setting and warrant greater widespread attention and future identification of strategies aimed at their reduction.

## Ethics approval

Ethical approval to conduct the study was granted by the Medical Research Centre of the Hamad Medical Corporation, Qatar (MRC-01–18-098). All study procedures and processes were conducted in accordance with that described in the ethics application and the guidelines and regulations outlined by the local medical research council.

## Consent to participate

Requirement for consent to participate was waivered by the approving ethics committee based on the nature and content of the study.

## Consent for publication

Consent for publication was sought and approved as part of the ethics approval.

## Data Availability

Data can be made available upon reasonable request to the corresponding author.
